# [Corrigendum] Hepatoprotective activity of chrysin is mediated through TNF-α in chemically-induced acute liver damage: An *in vivo* study and molecular modeling 

**DOI:** 10.3892/etm.2026.13068

**Published:** 2026-01-21

**Authors:** Anca Hermenean, Teodora Mariasiu, Inmaculada Navarro-González, Josefina Vegara-Meseguer, Eftimie Miuțescu, Sandipan Chakraborty, Horacio Pérez-Sánchez

Exp Ther Med 13:1671–1680, 2017; DOI: 10.3892/etm.2017.4181

Subsequently to the publication of the above article, an interested reader drew to the authors’ attention that histological data featured in Fig. 3 on p. 1675, and one of the transmission electron microscopy images shown in Fig. 6 on p. 1678, were strikingly similar to data that had already appeared in a pair of publications featuring the first author on the paper (A. Hermenean); moreover, the histological images shown in Figs. 4B and 5B also appeared to contain an overlapping section, suggesting that these were derived from the same original source where the results from differently performed experiments were intended to have been portrayed.

After having re-examined their original data, the authors were able to identify where errors had inadvertently occurred in the assembly of these figures. New versions of Fig. 3 (showing the results of a repetition of the Oil Red O staining experiments performed under identical conditions, which served to reconfirm the results and the corresponding statistical analysis), Fig. 4 (showing the correct results for the experiment shown in Fig. 4B), and Fig. 6 (showing the correct data for the experiment shown in Fig. 6D) are shown on the next three pages. Note that the revised data shown for these figures do not affect the overall conclusions reported in the paper. All the authors agree with the publication of this corrigendum; furthermore, they apologize to the Editor of *Experimental and Therapeutic Medicine* and to the readership for any inconvenience caused.

## Figures and Tables

**Figure 3:**
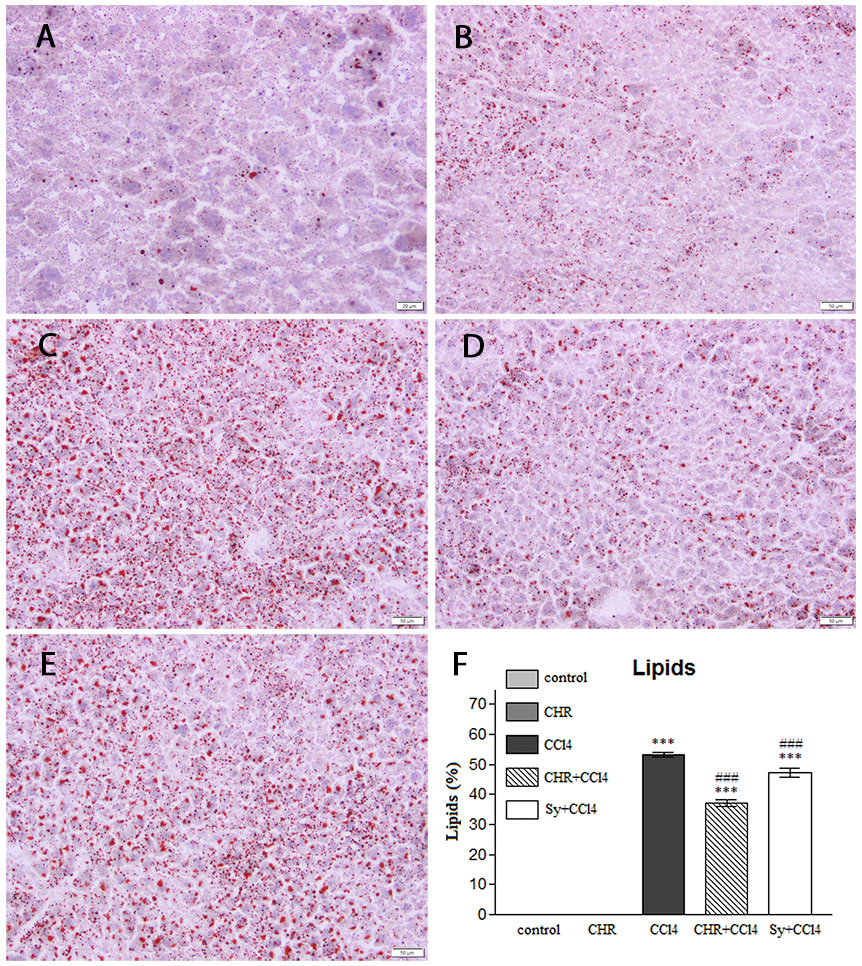
Effect of chrysin on lipid accumulation in the liver of CCl4-treated mice. Representative images of Oil Red O stained liver sections of the (A) control group, (B) CHR group, (C) CCl4 group, (D) CHR+CCl4 group and (E) Sy+CCl4 group. Lipid drops, indicated in red. Scale bar, 50 µm. (F) The percentage of lipids in mice liver samples. Results are represented as the mean ± standard deviation (n=5). Scale bar, 50 µm ^***^P<0.001 vs. control; ^###^P<0.001 vs. the CCl4 group. CCl4, carbon tetrachloride; CHR, chrysin; Sy, silymarin.

**Figure 4:**
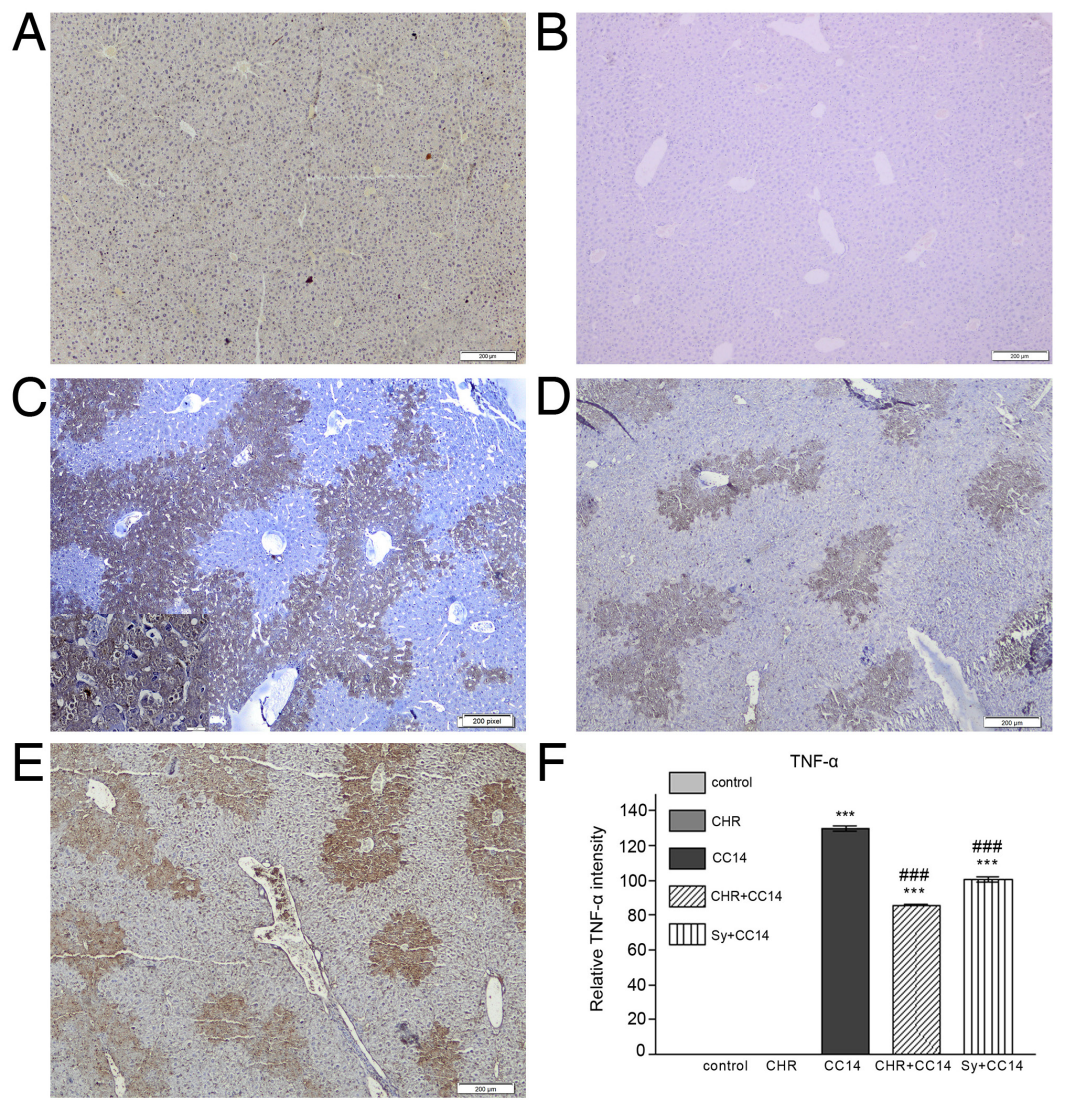
Effect of chrysin on the expression and distribution of TNF-α protein in the liver of CCl4-treated mice. (A) Control group, (B) CHR group, (C) CCl4 group, (D) CHR+CCl4 group and (E) Sy+CCl4 group. Scale bar, 200 µm. (F) Quantification of TNF-α staining intensity relative to control and chrysin groups. Results are represented as the mean ± standard deviation (n=5 mice per group). ^***^P<0.001 vs. the control group; ^###^P<0.001 vs. the CCl4 group. TNF-α, tumor necrosis factor-α; CCl4, carbon tetrachloride; CHR, chrysin; Sy, silymarin.

**Figure 6:**
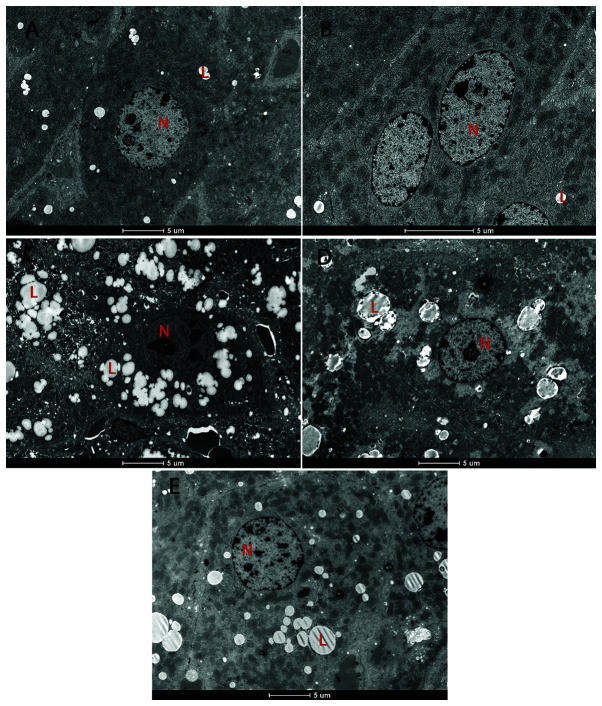
Effect of chrysin on hepatocyte ultrastructure in CCl4-treated mice. (A) The control group showed normal N and L. (B) CHR group. (C) The CCl4 group showed an edematous cytoplasm matrix with sER proliferation, and an increased quantity and size of L. (D) The CHR+CCl4 group showed a reduction in the quantity and size of L, and no sER proliferation. (E) The Sy+CCl4 group showed a reduction in the quantity and size of L, and no sER proliferation. Transmission electron miscopy images representative of the group. Scale bar, 50 µm. N, nucleus; L, lipid drop; sER, smooth endoplasmic reticulum.

